# National Insurance and the Voluntary Hospitals

**Published:** 1913-08-02

**Authors:** 


### NATIONAL INSURANCE AND THE VOLUNTARY HOSPITALS.

### The Hon. Harry Lawson Defends the Hospitals.

IMPORTANT AMENDMENT OF CLAUSE 12.

We take the following report from the *Daily Telegraph* on the proceedings at the meeting of the Standing Committee of the House of Commons on National Insurance held on July 29:—

An interesting discussion on the subject of insured persons and hospital treatment, with special reference to the question of contributions to the funds of institutions by persons receiving sick benefit for the period during which they are inmates, was the outstanding feature of yesterday's sitting of the Standing Committee of the House of Commons which is engaged upon the consideration of the National Insurance Act (1911) Amendment Bill. Mr. J. W. Wilson presided.

Mr. C. Bathurst moved to repeal Section 12 of the principal Act, which provides that no payment shall be made on account of sickness, disablement, or maternity benefit to or in respect of any insured person whilst an inmate of a workhouse, hospital, asylum, convalescent home, sanatorium, or similar institution maintained by a public authority, or out of public funds, or by voluntary subscriptions. The section enacts that in such case the benefit is to be devoted to the relief of the contributor's dependants, to the uses of the Insurance Committee, or, if there is an agreement between the society and the institution, to the institution in which the insured person is a patient.

The section, Mr. Bathurst said, was practically a dead letter and, where it was not, there was no uniformity in its administration as between the various societies and the Insurance Committees. It imposed on societies the necessity of finding some plausible excuse for paying the sick benefit in such a way that it would ultimately reach the sick person in the hospital. This part of the Act was being successfully evaded, and it was entirely contrary to the former practice of friendly societies, who paid full sickness benefit to their members when they were in hospital. Hospitals were largely supported by the voluntary contributions of workmen themselves. (Hear, hear.)

Mr. Goulding, supporting the amendment, said everybody agreed that the position at present was mischievous and chaotic.

### Government's Proposal.

Mr. Masterman (Financial Secretary to the Treasury) stated at some length the view of the Government. He thought the Committee should not decide to get rid of the whole of Section 12 in order to meet one point in connection with it. So far as insured persons who had dependants were concerned no question arose, as the sick pay went to the dependants when an insured person was in hospital. In the case of insured persons who had no dependants, at present it was optional to the societies to use the money which would have been their sick pay if they had not gone into hospital for any purpose which was for their benefit, and it was doubtful whether they could pay over any of the money in cash. There had been a great deal of difficulty in connection with the clause. The actuarial calculation on which the Bill was framed assumed that in every case an insured person without dependants would receive full sick pay in some form or other. He agreed with Mr. Bathurst that the time had come when they might clearly state in the Act that a man or woman without dependants should receive full sick pay in some form or other. (Cheers.)

He did not, however, want entirely to take away discretion from the societies. It was not always advisable, perhaps, when a man left a hospital after being there several weeks, to give him necessarily a lump sum of £5 or £10, but he should have the full amount of his benefit either in cash or in kind. Of course, it might be argued that some of the money should go to the hospital. (Hear, hear.) If the insured person was willing, some of the money could go to the hospital, but he (Mr. Masterman) would be very chary of making an arrangement by which the hospitals should receive all the money. That would raise the whole question of voluntary hospitals in a form least favourable to those hospitals.

### Practice of Hospitals.

They had not taken paying patients from among the poor, and he had received no request from them that they should take such patients. If they did so he had no doubt that there would be a great decrease in their subscriptions in other directions. No pressure ought to be put on patients to sign away their sick pay to hospitals. (Hear, hear.)

Instead of repealing Section 12, he proposed to meet the difficulty by inserting the following new clause:

Section 12 of the principal Act shall have effect as though the first proviso to Sub-section 2 of that section were omitted therefrom, and any sum which, but for the provisions of that section, would have been payable to any person on account of sickness, disablement, or maternity benefit, if and so far as it is not paid or applied in accordance with the provisions of that section while the person to or in respect of whom it would have been payable is an inmate of any workhouse, hospital, asylum, convalescent home, or infirmary, shall, if the society or committee administering the benefit thinks fit, be applied in the provision of any surgical appliance required for the person or otherwise for his benefit, after he ceases to be an inmate, or, if it is not so expended, shall be paid in cash to the person after leaving the institution in a lump sum, or in instalments, as the society or committee thinks fit.

Mr. Worthington Evans remarked that some approved societies were paying the money to insured persons without dependants who were in hospital and taking the risk, whilst others were not. Clearly, there should be one rule for all societies. (Hear, hear.) He knew of a case where a girl had received only one week's sickness benefit out of nine because for eight weeks she was in hospital, where she paid 5s. a week for maintenance.

### Effects of the Act.

Mr. Lawson did not think that the Government appreciated in the least the position of the great hospitals. Two years ago, when the National Insurance Bill was under discussion, a strong protest was made in the House of Commons on behalf of the hospitals, and everything that was said by those who could speak for the hospitals had turned out to be absolutely true. (Hear, hear.) A broad line must be drawn between the provincial hospitals and the London hospitals. In the case of the former 60 or 70 per cent. of the cost of maintenance was furnished by working men through collections in mills and works, whereas in London only from 10 to 12 per cent. was paid by the working classes, the remainder coming from the voluntary subscriptions of the well-to-do. Under the Act additional work had been thrown on the hospitals in connection with the more serious cases. There had been a slight falling off in the out-patients' department, but the costly part of hospital work had grown in amount and in expense.

Subscriptions had fallen off. Letters were received every day by hospital secretaries from employers of labour saying that as they paid insurance they could not continue their subscriptions, or could not continue them to the same amount. The quinquennial subscription list of the great hospital with which he was connected—the London Hospital—had been very much smaller than was ever known before. That was only an example. The Financial Secretary to the Treasury said it would be a great mistake for hospitals to accept payment for the services they rendered out of the insurance money of the working people.

Mr. Masterman: I think I said to impose payment.

Mr. Lawson observed that, anyhow, the right hon. gentleman seemed to wish to make it a little more difficult than before for hospitals to obtain from the insurance money any subscription which would, to some extent at least, cover the cost of treatment.

Mr. Masterman: No, no.

### Example of the Provinces.

Mr. Lawson said that was what he wanted to guard against very strongly. The treatment was becoming much more costly to hospitals, and he did not know where the money was to come from unless the matter was faced from the insurance point of view. That had not been done. (Hear, hear.) It seemed to him as if the Committee were going to take a backward step in that respect. He did not believe in the danger of compulsion on the part of the hospital authorities, but he thought that if a workman received a lump sum after he had undergone the highest form of special treatment in a great hospital he ought to be under an obligation to pay part of it over to the hospital. (Cheers.) In the provincial hospitals the men were paying. In London it was entirely a matter of chance so far as the working man was concerned, except as regarded what was collected by the Hospital Saturday Fund.

He viewed with disfavour any amendment which would make the case of the hospitals more difficult. He did not want to see a rate in aid for the great hospitals. (Hear, hear.) Under Section 12 the Insurance Committee could make a payment if it thought fit, and it would be a strong order to deprive the hospital of this assistance.

Mr. Masterman: We do not propose to touch that power at all. If we do not delete Section 12 but accept my amendment that power remains as in the original Act.

Mr. Lawson said he would have preferred to see it strengthened. He was not afraid of the word "compulsion"; but he did not say it should be used. What he wanted was to make it more a normal matter for the money to be paid to the hospital which had given the special treatment. He warned the Committee that if because they wanted to hand over—it was a very pleasant thing to do—a lump sum to the workman on his leaving any hospital they were going to decrease the resources of the great London hospitals, which really advanced the science of therapeutics upon which the health of the country depended, they would be doing a great dis-service to the working classes. (Cheers.)

Mr. H. W. Forster said that Mr. Lawson's view was worthy of consideration. Without wishing to impose charges on insured persons he thought they should be given some opportunity of contributing to the funds of the hospital whilst receiving treatment.

Mr. G. Roberts argued that any pressure upon a person to make a payment whilst in hospital would destroy the voluntary character of the hospitals and arouse a demand for public control.

Mr. Masterman said the friendly societies ought to be allowed to go on working the Act in connection with the hospitals before opening up the whole hospital problem. Instead of his amendment doing any injury to the hospitals, in so far as it would have any effect at all, he believed it would benefit them.

After some further discussion, the *Times* states the proposal to repeal Section 12 was defeated by 30 to 15, and Mr. Masterman's amendment, after protest from several members against the manner in which it had been forced upon the Committee without notice, was afterwards agreed to as a new clause.

